# Entrapment of Amphipathic Drugs in Core–Shell Polymeric Nanoparticles under Batch Conditions—The Role of Control and Solubility Parameters

**DOI:** 10.1021/acs.langmuir.4c02721

**Published:** 2024-09-24

**Authors:** Łukasz Lamch, Rafał Szukiewicz

**Affiliations:** † Department of Engineering and Technology of Chemical Processes, Faculty of Chemistry, Wrocław University of Science and Technology, Wybrzeże Wyspiańskiego 27, Wrocław 50-370, Poland; ‡ Faculty of Physics, Institute of Experimental Physics, University of Wroclaw, Maxa Borna 9, Wroclaw 50-204, Poland

## Abstract

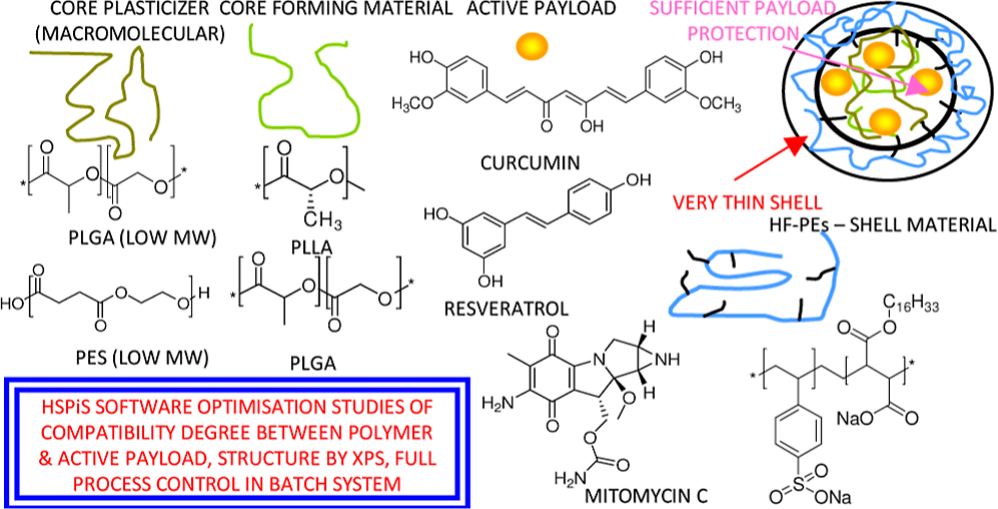

The amphipathic bioactive compounds curcumin, resveratrol, and mitomycin C, which have similar solubility parameter component distributions, have been studied for encapsulation under batch conditions into core–shell nanocarriers composed of external hydrophobically functionalized polyelectrolytes and an inner matrix of polyesters or polyester blends: poly(l-lactide), poly(lactide-*co*-glycolide), and/or poly(ethylene succinate). Our contribution comprises determining the influence of process parameters on the properties and quality of the final products, namely core–shell nanoparticles loaded with appropriate drugs, according to process analysis technologymanagement. The crucial roles of the organic phase dosing rates and process temperatures were carefully investigated. Moreover, a technically feasible method of removing organic solvents from aqueous dispersions—stripping with inert gas—was employed and evaluated via FT-IR studies. The experiments were supported by the calculation and analysis of solubility parameters (δ) and dispersion (δ_d_), polar (δ_p_), and hydrogen bond (δ_h_) components utilizing HSPiP software. The payload locus and sample morphology were studied via atomic force microscopy and X-ray photoelectron spectroscopy analyses with Ar^+^ sputtering. It was demonstrated that dosing rates of organic phases not exceeding ca. 0.5 mL/min per 1 L of aqueous dispersion of hydrophobically functionalized polyelectrolytes made it possible to obtain core–shell nanoparticles of ca. 100–150 nm with a very narrow polydispersity (PdI < 0.2). The locus of amphipathic payloads in nanocarriers, mostly within the core polymeric structure, was in good agreement with the results of solubility parameter component studies: water-insoluble polyesters with both polar and nonpolar interactions between chains serve as good host materials for amphipathic drugs.

## 1 Introduction

Numerous bioactive compounds possess amphipathic or weakly hydrophilic/hydrophobic characteristics, i.e., their structures contain both polar and nonpolar chemical motifs. Despite benefits in various applications—such compounds may be dispersed in both oil and aqueous phases—these structures are prone to undergo unwanted processes, such as chemical degradation, aggregation, or reversible changes in the intended (micro)environment, leading to loss of bioactivity.^1,2^ It should be noted that the aforementioned processes may occur in both polar and nonpolar surroundings, so it is difficult to design and prepare appropriate carrier systems that provide both physical and chemical stability to amphipathic molecules.^3^ The main problem may be inappropriate locus of the payload with respect to the host molecules, i.e., bioactive compounds adsorbed at the nanocarrier surface may be exposed to water molecules, resulting in deactivation or premature release.^4,5^ Therefore, it is desirable to design carrier systems comprising at least two microenvironments of differing character: internal hydrophobic and external hydrophilic microenvironments. Preferably, bioactive molecules should be incorporated within the inner structure, known as the core, which is surrounded by a polar core or corona. Two types of such nanocarriers have gained particular attention: polymeric micelles and core–shell nanoparticles.^4^ It should be noted that the internal cores of both types of nanocarriers may be composed of similar materials—hydrophobic polymers, such as polyester—and may be in a rubbery, glassy, or even liquid state to provide an appropriate amount of space for the guest molecules. The driving force for core–shell entrapment may arise from both the extremely low interfacial tension between organic and aqueous phases, controlled by the amphipathic compounds, and the chemical instability due to limited solubility of the drug and hydrophobic polymer in aqueous systems. Both processes lead to formation of core–shell nanoparticles since the amphiphile is adsorbed at the interface between the hydrophobic core and aqueous continuous phase.^1,2^

There is a general question as to how hydrophobicity, hydrophilicity, or amphipathicity may be quantitatively described. There are numerous indirect descriptors of these parameters, such as log (*P*), the electric dipole moment, the wetting angle, and the hydrophobic-liophilic balance (HLB). All of them may be determined experimentally [e.g., log (*P*) is defined as the partition of a particular substance between water, a model polar solvent, and n-octanol, a reference nonpolar solvent] or theoretically calculated (e.g., dipole moments may be calculated from electronic structure theory).^3,6,7^ The main drawback of these descriptors is the use of only one number—sometimes positive, negative, or equal to zero—for a particular molecule, regardless of its chemical structure, molecular weight, and the additive, synergistic, or subtractive contributions of particular chemical groups. Therefore, more accurate descriptors of hydrophobicity, hydrophilicity, and amphipathicity should include more than one number and more complex methods for comparing two or more molecules.^6,7^

To address the drawbacks of using a single number, descriptors of molecular character concepts based on cohesive energy have been introduced. In the simplest description, if the difference between the two substances’ cohesive energies is sufficiently low, they may be mutually soluble or miscible. This theory seems to be much more precise in the prediction and description of numerous systems, including polymers, dyes, drugs, coatings, and fibers, than parameters such as log (*P*), dipole moment, or contact angle.^7,8^ It should be noted that an appropriate border value (i.e., the critical value of the cohesive energy difference between the solubility and insolubility) must be known and approximated for a particular system. The total cohesive energy difference, most often approximated by the difference between the solubility parameter and the square root of the total cohesive energy divided by the molar volume, does not indicate the exact nature of the aforementioned interactions between two or more substances. Therefore, solubility parameter components, namely, the dispersion (δ_d_), polar (δ_p_), and hydrogen bond (δ_h_) components, have been introduced, and the square root of the sum of squares of those numbers constitutes the total solubility parameter (δ)
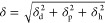
1

To calculate the solubility parameter difference (Δδ), a parameter that quantitatively describes the compatibility between two substances, denoted with numbered superscripts (1 and 2), e.g., polymers/plasticizers, polymers/drugs, or polymers/solvents, we may use the following formula
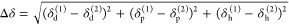
2

It should be noted that, according to particular theories, appropriate squares describing differences in dispersion and polar and hydrogen bonding interactions may be divided by appropriate constants to take into account the weighted influence of particular forces. It should be emphasized that the aforementioned theory may predict numerous process parameters, especially in the field of optimal solvents or their mixtures and concentrations of organic phases, but cannot adequately describe kinetics.^6−9^ Therefore, numerous process parameters, especially dosing rates and temperatures, should be optimized experimentally, preferably utilizing scalable equipment resembling the designed industrial vessel.

The upscaling of processes that result in the formation of a colloidal dispersion is an emerging problem in numerous industries, including chemical, pharmaceutical, metallurgical, and so on. It should be emphasized that such processes require very careful control of the process parameters and are sensitive to even slight changes, e.g., in the stirring speed, vessel/system geometry, and temperature.^9,10^ The preparation of polymeric nanoparticles as host materials for bioactive compounds is especially important; such materials are very prone to chemical degradation or irreversible changes in physical structure in response to temperature, pH, or polarity variations. For small quantities of such nanocarriers, suitable and reproducible methods that provide nanoparticles with highly uniform dimensions and shapes can be used to construct microfluidic devices. On the other hand, batch or continuous-flow preparation of some nanocarriers may be performed by membrane-assisted processes, although this approach is restricted by ambient temperature and the availability of readily soluble building blocks and payloads. The limited applicability of microfluidic or membrane-assisted methods suggests the possibility of using multifunctional vessel-type equipment for nanocarrier preparation. Such equipment enables the precise control of temperature, stirring, and dosing rates as well as permits timely sampling (*online* control) or stripping with inert gas—all methods compatible with scalable industrial approaches. The whole philosophy, known as process analytical technology (PAT), constitutes basic methods in the pharmaceutical and fine chemical industries, making it possible to determine which parameters have the greatest impact on the final product quality.^11−20^

We chose three multifunctional drugs, curcumin (CUR), resveratrol (RES), and mitomycin C (MMC), with very high therapeutic potential (as anticancer, antimicrobial and/or anti-inflammatory agents) and similar amphipathic [weakly hydrophilic for MMC – log (*P*) = −0.4 or hydrophobic for CUR – log (*P*) = 3.29 and for RES – log (*P*) = 3.4] character with a high tendency to interact by dispersion (London) and hydrogen bonding forces. To ensure appropriate compatibility between the polymer matrix and the host drug, we selected the polyesters poly(l-lactide) (PLLA), poly(lactide-*co*-glycolide) (PLGA), and poly(ethylene succinate) (PES) as major building blocks and macromolecular plasticizers. Moreover, all of those drugs undergo gradual deactivation in aqueous systems through different pathways, involving aggregation or reversible chemical changes to CUR and RES as well as irreversible chemical degradation of MMC. Considering our previous studies and the usefulness of the methodology, we decided to encapsulate the drugs in core–shell nanoparticles, providing a convenient and scalable approach for addressing the hydrophobicity of the drugs. The careful design of the nanocarrier system must be followed by appropriate investigations of the preparation conditions as well as analysis of whether the desired localization of the bioactive compound within the nanocarrier structure (encapsulation within the core or anchoring to the external shell layer) has been achieved. Typically, core structures are better sites for poorly water-soluble materials whereas charged shells may incorporate hydrophilic payloads, although there are numerous exceptions, especially those related to stability and the ability to undergo controlled (e.g., physically or chemically triggered) gradual degradation of the polymer.

The main aim of the present research was to demonstrate the scalability and flexibility of core–shell encapsulation in polyester nanoparticles stabilized by hydrophobically functionalized polyelectrolytes (HF-PEs) of both naturally occurring drugs, curcumin (CUR)^9^ and resveratrol (RES),^21^ and the biotechnologically produced mitomycin C (MMC).^10^ The batch-type core–shell entrapment technique^9,10,21^ constitutes a fully scalable preparation method for core–shell nanoparticles, and analytical techniques [including atomic force microscopy (AFM) and X-ray photoelectron spectroscopy (XPS) with Ar^+^ sputtering] enable the determination of payload location as well as prove its chemical stability. The most important points of interest include (i) the choice of optimal nanocarrier structures for different payloads with similar amphipathic characteristics but differing chemical characteristics; (ii) careful studies of compatibility between core-forming polymers (PLLA, PLGA, and/or PES) and payloads (CUR, RES, or MMC); (iii) the use of batch-type vessels to optimize preparation conditions for core–shell nanoparticles with the desired properties, including the influence of temperature; and (iv) the characterization of the obtained nanocarriers to determine their morphologies and internal structures. Our research studies provide direct proof of core–shell structure of different carrier systems, thus enabling us to better understand their chemical structure and stability. The studies performed show a very high potential of this optimization methodology, involving not only theoretical calculations of payload-host polymer compatibility but also scalable preparation methodologies that meet known requirements.

## 2 Experimental Section

### 2.1 Materials

Hydrophobic polymers PLGA (*M*_w_ = 45 kDa; LA/GA = 50:50) and PLLA (*M*_w_ = 5 kDa) were purchased from Sigma-Aldrich (Burlington, MA, USA). CUR and RES were obtained from Archem while (MMC) was obtained from Sigma-Aldrich (Burlington, MA, USA). HF-PEs and low molecular weight PES were synthesized, purified, and characterized according to newly devised methods described in detail previously.^20,22,23^ The structures and brief descriptions of the studied materials are listed in Table 1. All solvents were of analytical grade and purchased from Avantor Performance Materials (Gliwice, Poland). Milli-Q purified water was used in all the experiments.

**Table 1:**
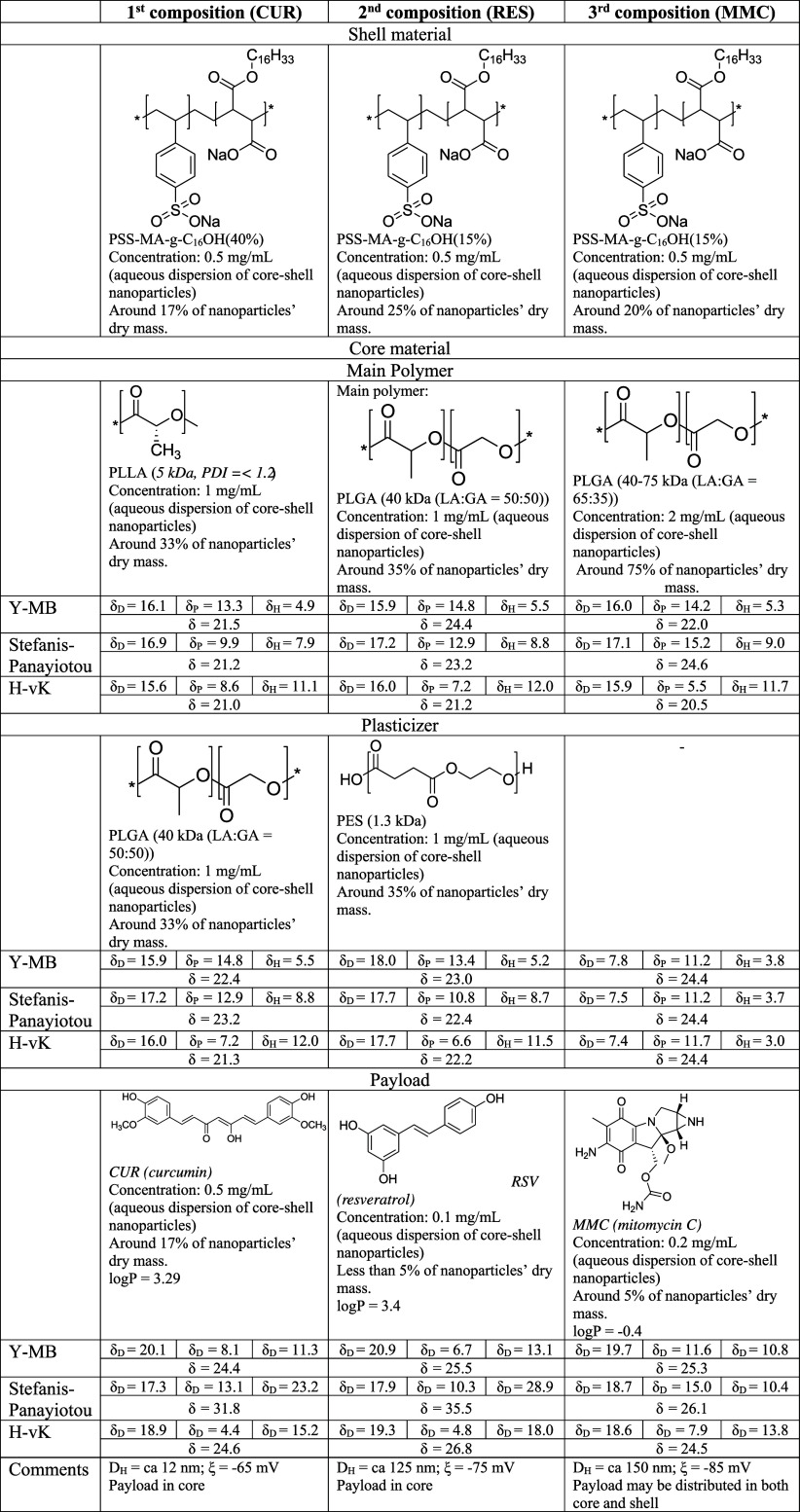
Structures and Properties of the Components of Core–shell Nanoparticles, Loaded with Curcumin, Resveratrol, and Mitomycin C

### 2.2 Hansen Solubility Parameters

Hansen solubility parameters and their components (δ_d_, δ_p_, and δ_h_) of London dispersive, polarity, and hydrogen bonding molecular interactions may be estimated utilizing group increment methods, assuming an additive effect of particular chemical motifs. Nowadays, such approaches may be easily automatized by appropriate computer programs. We estimated the parameters for each component using HSPiP software, as shown in Table 1, using the “DIY” menu. Taking into account the corrections for different molecular weights we standardized our calculations for low molecular weight compounds as well as polymers by using a single unit for calculations. Calculations were performed for three different models: Y-MB, Stefanis-Panayiotou, and Hoftyzer-van Krevelen.^6,7^ The group increments for Stefanis-Panayiotou and Hoftyzer-van Krevelen methods are provided by HSPiP software while the exact numbers of particular chemical motifs for Hansen solubility parameter components calculations were manually taken from the structures.

### 2.3 Experimental Setup

**Figure 1:**
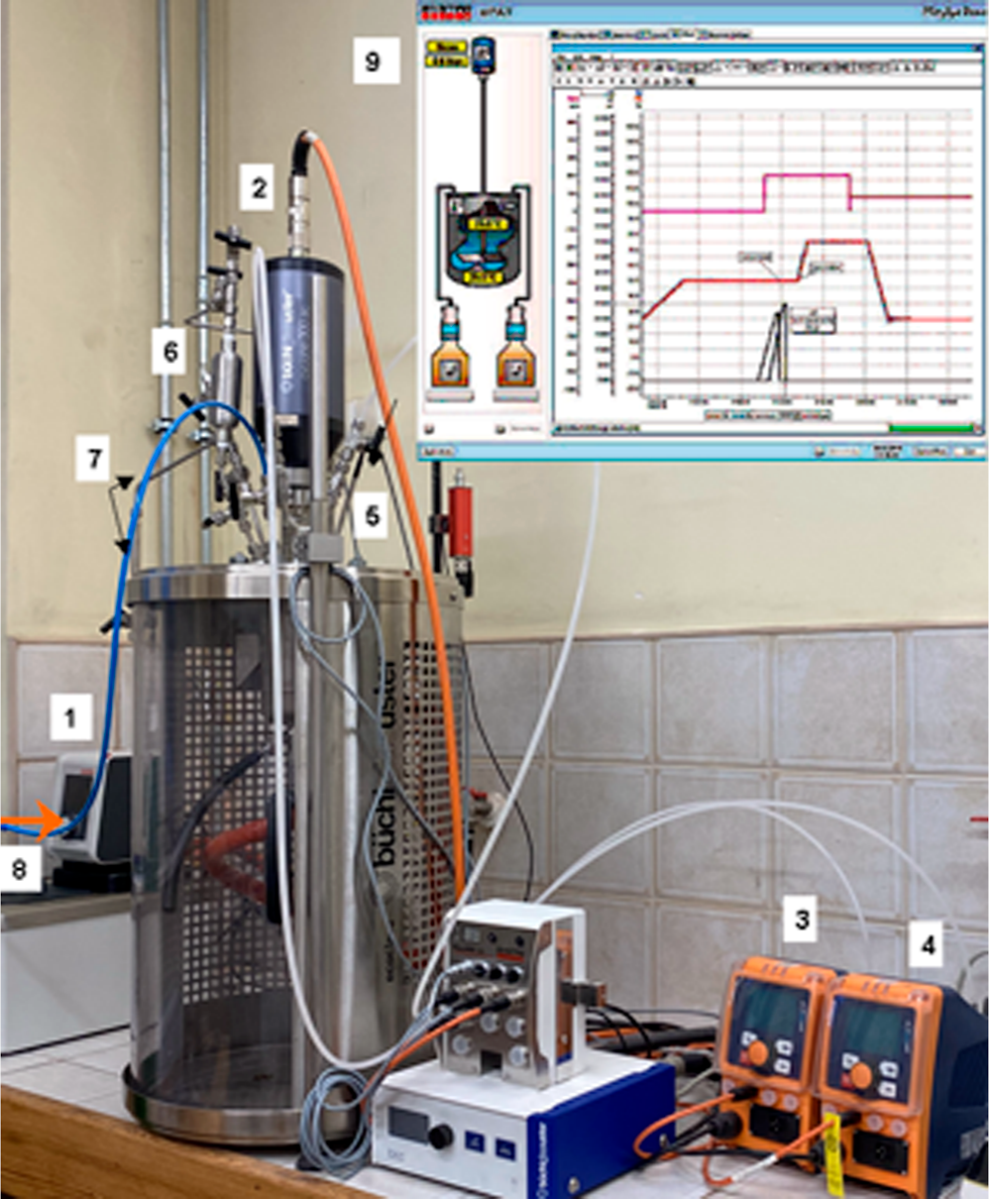
Vessel batch-type system, equipped with a thermostatic unit (1), stirrer (2), two gravimetric dosing systems (3 and 4), pH probe (5), pressure/gravitational burette up to 50 mL (6), pipette (volume of sample, 5 mL) with pressure reducing system and filter (7), inert gas purge (8), and ePAT control system (9).

The active payload core–shell entrapment was performed utilizing an Ecoclave pressure vessel (BuchiGlasUster, Switzerland) equipped with an external thermostatic unit—see no. 1 in Figure 1—(Julabo, Germany), a mechanical stirrer—see no. 1 in Figure 1—and two independent gravimetric dosing systems—see no. 3 and 4 in Figure 1—as well as a pH meter—see no. 5 in Figure 1. The system enables a single dose of a substance of up to 50 mL via a gravitational or pressure burette—see no. 6 in Figure 1—as well as timely sampling of the mixture inside the vessel utilizing a pipette equipped with a filter and pressure reducing system (fixed volume, 5 mL; see no. 7 in Figure 1). The pH of the solution inside the vessel was adjusted to 7 ± 0.5 by 1 M HCl or 1 M NaOH (dosage via a gravimetric system). In order to assume nonoxidative conditions of all experiments, the solvent removal system was purged by continuous N_2_ stripping—see no. 8 in Figure 1 (inert gas inlet). All parameters were controlled by an ePAT automatization system (SYSTAG, Switzerland)—see insert no 9 in Figure 1.

### 2.4 Preparation of Drug-Loaded Core–Shell Nanoparticles under Batch Conditions

Briefly, appropriate amounts of polymers were dissolved in an acetone (pure PLGA) or THF/acetone mixture (1:1, *v*/*v*; blended polymers) at a total concentration of 10 mg/mL. Appropriate drugs were dissolved in organic (for CUR and RES) or aqueous solution (for MMC). To optimize the preparation of core–shell nanoparticles, which are stabilized by HF-PE, different conditions were tested. The equipment elements are illustrated and numbered 1 to 8 in Figure 1.

For preparation of the CUR-loaded nanoparticles, the vessel was filled with 200 mL of Milli-Q water and heated to 85 °C (controlled by a Pt100 via thermostatic unit 1 and control system 9). Water inside the reactor was continuously purged with N_2_ (see 8 in Figure 1) in order to assume further removal of organic solvent, and the system was kept under continuous stirring (stirrer 2, 850 rpm). When the temperature was stable, a solution of PSS-MA-*g*-C_16_OH (40%) in water (2.5 mg/mL, 50 mL) was instantly introduced via a burette (6) while both dosing lines (3 and 4) were filled with an organic solution and an aqueous 0.1 M NaOH, respectively. The dosing rate for line 3 was set as 0.695, 0.139, or 0.104 mL/min (0.570, 0.118, or 0.086 g/min, respectively, total mass 42.5 g) while aqueous sodium hydroxide (line 4) was introduced into the vessel only if the pH (probe 5) decreased below 6.5 (the total volume of the added 0.1 M NaOH was lower than 2 mL). Following the whole organic phase introduction, a sample of solution (5 mL, see pipette 7) was taken every 10 min to determine whether organic solvents are still present in the solution. Finally, the dispersion of nanocarriers was transferred from the vessel via the bottom valve into a beaker, followed by rapid cooling by immersing in ice mixed with cold water.

For the preparation of the RES- and MMC-loaded nanoparticles, the vessel was filled with 250 mL of PSS-MA-*g*-C_16_OH (15%) solution in Milli-Q water. The solution inside the reactor was continuously purged with N_2_ (see 8 in Figure 1) in order to assume further removal of organic solvent, and the system was kept under continuous stirring (stirrer 2, 850 rpm). One dosing line (3) was filled with an appropriate organic solution, while the second (4) with aqueous 0.1 M NaOH. The dosing rate for line 3 was set as 0.695, 0.139, or 0.104 mL/min (0.545, 0.118, or 0.082 g/min, respectively, total mass 42.5 g) while aqueous sodium hydroxide (line 4) was introduced into the vessel only if the pH (probe 5) decreased below 6.5 (the total volume of the added 0.1 M NaOH was lower than ca. 5 mL). Following the whole organic phase introduction, a sample of solution (5 mL, see pipette 7) was taken every 10 min to determine whether organic solvents are still present in the solution. Finally, the dispersion of core–shell nanoparticles was transferred from the vessel via the bottom valve (not visible in Figure 1).

In order to remove any unencapsulated drug or precipitated polymer in its bulky form pipette 7 was equipped with metal frit while dispersions of core–shell nanoparticles, transferred by the bottom valve, were subsequently filtered via a porous membrane (pore sizes −0.45 μm). It should be emphasized that the concentration of solid core–shell nanoparticles in the final colloidal dispersion (ca. 2 mg/mL) is close to the limit of organic hydrophobic polymer fraction in an aqueous system.

### 2.5 Characterization of Core–Shell Nanoparticles

In order to determine colloidal properties, i.e., the size and its distribution, given by hydrodynamic diameter (*D*_H_) and polydispersity index (PdI) as well as zeta potential (ξ) of the obtained nanocarriers, dynamic light scattering (DLS) technique was utilized (Ultra Red Instrument, Malvern Instruments, UK with a 10 mW He–Ne laser, λ = 633 nm). The detection mode accessible for angle 173, (i.e., noninvasive backscattering), 90, or 13° was automatically chosen for each measurement. Each measurement was performed in triplicate, and the results for intensity mean hydrodynamic diameter (*D*_H_), polydispersity index (PdI), or zeta potential (ξ) are given as the average with standard deviation.

The percentage drug loading (DL %) and the encapsulation efficiency (EE %) were calculated utilizing the absorbance spectra of CUR, RES, and MMC recorded by spectrophotometric measurements in the range of 200–600 nm with the use of a UV-3600 (Shimadzu) double beam spectrophotometer with a 0.5 cm quartz cuvette. Each sample was diluted 5-fold with acetone or distilled water prior to the measurement. Prior to determining the MMC in the core–shell nanoparticles’ dispersions, the samples were centrifuged, and analysis was performed for the nonencapsulated drugs. The absorbance was recorded at 326 nm, and a molar extinction coefficient of 30,000 dm^3^ mol^–1^ cm^–1^ was used for the calculations. The DL % and EE % values were calculated according to eqs 3 and 4, respectively.
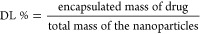
3
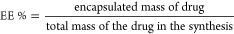
4

### 2.6 XPS Studies with Ar^+^ Sputtering

The surface chemical composition of the synthesized materials was investigated by XPS. A nonmonochromatized X-ray source with a Mg anode lamp (with a Mg-Kα radiation source, 1253.6 eV) was used in all analyses. High-resolution photoelectron energy spectra were recorded with an AES/XPS system EA10 (Leybold-Heraeus GmbH, Cologne, Germany) at room temperature. The pressure in the chamber during the measurements was less than 5 × 10^–8^ mbar. The spectrometer energy scale was calibrated using Au 4f, Ag 3d, and Cu 2p lines, and all of the acquired spectra were calibrated to the adventitious carbon C 1s line at 285 eV. The overall resolution of the spectrometer during the measurements was estimated to be 0.96 eV according to the full width at half-maximum (fwhm) of the Ag 3d_5/2_ line. WSpectra version 8 (R. Unwin, 2001) data acquisition software was used to collect the data, and CasaXPS version 2.3 (Casa Software Ltd., Teignmouth, UK) was used for data processing (deconvolution). To determine the chemical composition of the surface, first, a wide-range energy scan was performed on the basis of which detailed (high-resolution) scans of the element emission lines were then performed. The concentration of atoms in the sample was determined on the basis of XPS spectral analysis, taking into account the presence of individual elements.

The ion etching process was carried out sequentially using an IS 40C1 ion gun from the PREVAC Company with the following operating parameters: ion current, 2 μA/cm^2^; ion accelerating voltage, 1 kV; argon pressure, 5 × 10^–5^ mbar; and distance from the sample, 30 mm. The etching sequence was carried out until a signal from the core was obtained.

The colloidal dispersions were diluted 4-fold, dropped onto aluminum plates, and allowed to adsorb on the surface, followed by water evaporation at room temperature. The samples were analyzed prior to etching and after specified periods of Ar^+^ sputtering. AFM images were obtained using the same solution dropped onto mica pieces and after drying analyzed by AFM using an NaioAFM from the Nanosurf company in the tapping mode with a standard cantilever. The images were analyzed with WSxM software.

## 3 Results and Discussion

### 3.1 Preparation of Nanocarriers under Batch Conditions

In general, the design of an appropriate carrier system for particular bioactive payload needs careful choice of not only building blocks, e.g., hydrophobic/amphiphilic polymers and plasticizers, but also preparation conditions. Typically, all preparation steps (dissolution of polymers/payloads in appropriate solvents, organic and aqueous phases mixing, organic solvent removal, etc.) are preformed in ambient conditions (temperatures between 5 and 37 °C, atmospheric pressure) in order to avoid chemical changes of bioactive payload.^4,10^ On the other hand, such procedures, executed at ± room temperature may lead to suboptimal values of DL %, i.e., situations, when the active payload comprise only a few percent of the whole carrier by mass.^9^ Therefore, sometimes it may be needed to use elevated temperatures, e.g., slightly lower than boiling point of water, followed by shock cooling in order to obtain nanoparticles with a “frozen” state polymer matrix.^20^ For our studies, the dosing rate of the organic phase was the key factor controlling particles sizes, while other process parameters, like temperature, stirring rate, and concentrations of amphiphilic (i.e.,HF-PE) and hydrophobic polymers as well as the payload were carefully studied in our previous papers.^9,10,20^ Such assumption was also supported by the key role of local supersaturation, leading to increased nucleation rates and formation of nanoparticles.^1,2^ It should be emphasized that any computational methods may be very helpful in finding optimal composition and conditions, especially simplified methods using group increment methods.

#### 3.1.1 Compatibility Studies by Hansen Solubility Parameters

The polymer matrix composition was optimized by utilizing the solubility parameters of both the drug and the host polymers. Historically, numerous approaches are available for obtaining solubility parameters and their dispersion (δ_d_), polar (δ_p_), and hydrogen bond (δ_h_) interaction components: thermodynamic measurements and/or investigations of affinity to given stationary phases via gas chromatography (direct experimental methods);^6^ statistical analysis of the experimental data of solubility in at least ca. 20 organic solvents (denoted as “0”—insoluble and “1”—soluble; accuracy depends on the choice and number of organic solvents);^7^ group contribution methods (assuming that the influence of particular common chemical groups or motifs is additive and independent of neighboring groups);^6,7^ and molecular modeling approaches (utilized especially for oligomers and hydrophilic/amphiphilic polymers).^24^ It should be emphasized that the values of solubility parameters and, especially, of their components may differ significantly depending on the method used; in particular, values calculated according to Hoy’s group increment method cannot be compared with those obtained by any other approach.^3,24−30^ To obtain the most reliable data, the compared values of solubility parameters and their components should be calculated or measured utilizing the same method.^24^ One of the first commonly used approaches utilizing the group increment method was the calculation of cohesive energy by Fedors, which enabled determination of the total solubility parameter (δ) using a limited number of chemical groups and atoms. Later, two different methods that are incompatible with each other, Hoy’s and Hoftyzer-van Krevelen’s methods, were developed; these methods enable the calculation of dispersion (δ_d_), polar (δ_p_), and hydrogen bond (δ_h_) component contributions to the solubility parameter, utilizing variably accurate values for particular chemical groups (typically, errors for nonpolar fragment were less significant).^6,7^ The development of computational methods enabled the development of better approaches comprising basic (1st order) and secondary (2nd order) groups or substructures (method by Stefanis-Panayiotou) as well as allowing the calculation of parameters (even with more or less accurate weight % of solute in saturated solution) from SMILES–Y-MB chemical structures by Hiroshi Yamamoto. Currently, all of the aforementioned approaches are incorporated into HSPiP software, and the (generally) most accurate Y-MB method is considered the basic one.

The compositions and selected properties of the “building blocks”, i.e., stabilizing HF-PEs as the shell material, the core-forming polyester/polyester-type plasticizers, and the payloads (CUR, RES, and MMC) are shown in Table 1. Notably, all of the values of the solubility parameters (δ, so-called total solubility parameter) and their dispersion (δ_d_), polar (δ_p_), and hydrogen bond (δ_h_) components were assessed utilizing HSPiP software via appropriate group increment methods. It is worth noticing that all the utilized methods (Y-MB, Stefanis-Panayiotou, and Hoftyzer-van Krevelen’s) are still in use due to different “emphasize” in calculation of particular components (δ_d_, δ_p_, and δ_h_), although such division of solubility parameter is considered to be arbitrary but very useful from the experimental point of view.^3,6,7,24−30^ Therefore, our studies comprise use of three different methodologies in order to avoid misunderstanding, which is possible to occur, when only one approach is implemented for calculations.

The calculated values of δ_d_, δ_p_, and δ_h_ components for hydrophobic polymers/oligomers, although different for the particular calculation method, clearly show that polyester-type compounds exhibit simultaneously strong London (δ_d_ values of ca. 15.5–21 MPa^0.5^) and polar-type (sum of δ_p_ and δ_h_ values between 16 and 20 MPa^0.5^) interactions of nearly equal partition. It should be emphasized that both polar and hydrogen-bonding components represent very similar types of interactions, connected with polar chemical motifs, so particular models may significantly differ in this case. Bioactive payloads exhibit very similar behavior—high values of both London (δ_d_ values of ca. 17.5–21 MPa^0.5^) and polar-type interactions (sum of δ_p_ and δ_h_ values between 19 and 22 MPa^0.5^)—although Stefanis-Panayiotou’s model indicates ca. two times higher sum of δ_p_ and δ_h_. Such very rough considerations indicate that our polymers/oligomers and their blends may constitute a good host material for encapsulation of CUR, RES, and MMC. In order to prove our findings we have calculated (according to eq 2) values of solubility parameter difference (Δδ) between particular polymers/oligomers (in order to show possibility of their blend formation) and payload–host material pairs (to determine if a particular polymer may be compatible with the desired bioactive payload)—see Tables S1–S3 in Supporting Information. In general, values of Δδ were very low (<5 MPa^0.5^) for any model (Y-MB, Hoftyzer-van Krevelen’s, and Stefanis-Panayiotou’s) and pair of polymers/oligomers (PES-PLLA, PLLA-PLGA, and PES-PLGA), clearly showing that one compound may play the role as plasticizer to each other. Moreover, each model indicates that MMC exhibits high compatibility (values of Δδ < 7.5 MPa^0.5^) with all polymers/oligomers so any single one or blend may be used for its encapsulation. The behavior of CUR and RES significantly differs for various methods, although all models indicate that RES is the less compatible payload (values of Δδ between 7.5 and 21 MPa^0.5^). Taking into account that the Y-MB model is considered to be the most reliable one, PLLA-PLGA and PLGA-PES blends may play a role as host material for CUR and RES, respectively, since values of Δδ are between 8 and 12.5 MPa^0.5^ while systems with Δδ > 15 MPa^0.5^ are said to exhibit complete lack of compatibility.

#### 3.1.2 Influence of Organic Phase Dosing Rates on Product Quality

The formation of appropriate nanocarrier is ruled not only by the thermodynamics, generally accurately approximated by solubility parameters’ studies, but also by kinetics. Taking into account typically the ionic character of stabilizers, e.g., surfactants, polyelectrolytes, or their derivatives, the description by solubility parameters would be very inaccurate. Therefore, it is needed to optimize kinetics, especially in the fields of concentrations and dosing rates of particular phases, in an experimental way. A convenient equipment for such optimization studies constitute vessel-type batch systems with *in-line* pH control, stirrer, heating jacket, purge gas inlet/outlet as well as possibility to take out samples during the process.^10−18,31^ It should be emphasized that such an equipment is typical in the pharmaceutical industry and enables flexibility when switching between different products.

Despite the differences in methods (temperature, dosing rates, solvents used, etc.) and core compositions (PLGA, plasticized PLGA, or PLLA), the nanoparticles were characterized by mean diameters between 120 and 200 nm. It should be noted that the standard deviation (calculated for hydrodynamic diameter) for each sample is 1 or 2 orders of magnitude lower than the value and (typically) does not exceed ca. 5 nm, i.e., is comparable with the measurement accuracy. Moreover, the narrow size distribution is confirmed by low polydispersity indices, not exceeding ca. 0.2, i.e., within the range acceptable for nearly uniform or moderately polydisperse samples. In general, there were no significant differences between those values and those obtained in our previous studies utilizing the small-scale “beaker” method or membrane-assisted core–shell entrapment.^9,10^ This result clearly shows that the full scalability of the utilized methodology is independent of the type of system used and, generally, is connected to the optimized composition. For Hansen solubility parameter calculations and studies, see the subchapter “compatibility studies by Hansen solubility parameters”.

It should be noted (see Table 2 for details) that the fastest dosing rate of the organic phase (i.e., 0.695 mL/min by volume) generally resulted in the lowest EE %/DL % as well as the largest nanoparticles with significantly greater polydispersity. This difference is most likely caused by insufficient time for HF-PE to firmly adsorb on the nanoparticle surfaces to enable stabilization by electrostatic repulsion (note that the zeta potential ranged from −65 to −85 mV).^10,31−36^ This effect is very slight for MMC-loaded samples (MMC1–MMC3) because of the hydrophilic character of the drug molecules (the payload, partially adsorbed at the interface, may serve as an additional stabilizer). On the other hand, excessively slow dosing rates of the organic phase may have a negative influence on the sample EE %/DL % of CUR-loaded nanoparticles produced by utilizing a high-temperature process. It is likely that partial hydrolysis of HF-PE during long heating (ca. 7 h at 85 °C) is followed by insufficient adsorption onto nanoparticles formed during the last hours of the process.

**Table 2:** Synthesis Conditions and Characteristics of the Studied Core–Shell Nanoparticles^a^

abbr	HF-PE	payload	organic phase flow	*D*_H_ ± SD [nm]	PdI ± SD	*c*_pay_ [μM]	*c*_pay_ [mg/mL]	EE [%]	DL [%]
CUR1	PSS-MA-*g*-C_16_OH(40%)	CUR	0.570 g/min (0.695 mL/min)	245.1 ± 8.21	0.202 ± 0.035	325.8 ± 27.15	0.120 ± 0.010	23 ± 2	4.0 ± 0.33
CUR2	PSS-MA-*g*-C_16_OH(40%)	CUR	0.114 g/min (0.139 mL/min)	196.4 ± 1.15	0.089 ± 0.014	1384.4 ± 32.58	0.510 ± 0.012	97 ± 3	17.0 ± 0.4
CUR3	PSS-MA-*g*-C_16_OH(40%)	CUR	0.086 g/min (0.104 mL/min)	217.9 ± 5.05	0.187 ± 0.022	1140.1 ± 57.01	0.420 ± 0.021	80 ± 4	14.0 ± 0.7
RES1	PSS-MA-*g*-C_16_OH(15%)	RES	0.545 g/min (0.695 mL/min)	185.5 ± 2.51	0.109 ± 0.010	360.0 ± 39.43	0.105 ± 0.009	20 ± 2	3.5 ± 0.30
RES2	PSS-MA-*g*-C_16_OH(15%)	RES	0.109 g/min (0.139 mL/min)	143.1 ± 1.02	0.091 ± 0.008	670.3 ± 48.19	0.153 ± 0.011	29 ± 3	5.1 ± 0.37
RES3	PSS-MA-*g*-C_16_OH(15%)	RES	0.082 g/min (0.104 mL/min)	141.4 ± 2.37	0.121 ± 0.014	328.6 ± 83.24	0.075 ± 0.019	14 ± 4	2.5 ± 0.64
MMC1	PSS-MA-*g*-C_16_OH(15%)	MMC	0.545 g/min (0.695 mL/min)	133.5 ± 1.88	0.145 ± 0.012	403.8 ± 29.92	0.135 ± 0.010	26 ± 2	4.5 ± 0.34
MMC2	PSS-MA-*g*-C_16_OH(15%)	MMC	0.109 g/min (0.139 mL/min)	131.2 ± 2.15	0.133 ± 0.010	457.7 ± 23.93	0.153 ± 0.008	29 ± 2	5.1 ± 0.27
MMC3	PSS-MA-*g*-C_16_OH(15%)	MMC	0.082 g/min (0.104 mL/min)	135.8 ± 3.22	0.128 ± 0.011	430.8 ± 32.91	0.144 ± 0.011	27 ± 3	4.8 ± 0.37

a EE (%), encapsulation efficiency and DL (%), drug loading content.

#### 3.1.3 Role of Temperature in the Core–Shell Entrapment of Amphipathic Drugs

Typically, the formation of core–shell nanoparticles as colloidal carriers for bioactive substances is performed at room temperature to compensate for the lack of conditions favorable for the chemical degradation of the bioactive payload. In general, peptides, proteins, and other compounds that contain labile chemical bonds, e.g., esters or amides, are particularly susceptible to hydrolysis under acidic and/or basic conditions, especially at elevated temperatures. On the other hand, higher preparation temperatures enhance both solubility and polymer chain mobility, leading to better DL %, one of the most important parameters for any drug delivery systems. Therefore, optimizing the process temperature is one of the most challenging aspects of designing and preparing drug delivery systems. This step must take into consideration the following aspects: the temperature and/or pH favorable for degradation of the bioactive payload (i); the influence of temperature on drug solubility in both aqueous and nonpolar environments (ii); and the values of the transition temperatures of the host polymer(s), especially the melting point of the crystalline phase and the glass-transition temperature of the amorphous phase (iii). It should be noted that the latter two issues may be analyzed in terms of values calculated by HSPiP software.^6,7^

In general, two of the studied payloads, CUR and RES, are considered to be poorly water-soluble at neutral or acidic pH; their solubilities are equal to 0.002% (*m*/*m*) and 0.0013% (*m*/*m*) at 25 °C, respectively, and remain practically unchanged at temperatures between 25 and 90 °C. In order to compare solubility of CUR and RES in the polymer matrix we have calculated the predicted, according to HSPiP software, values of the drug mole fraction for RES-PLLA, RES-PLGA, CUR-PLLA, and CUR-PLGA systems. The maximal molar ratio in thermodynamically stable CUR-PLLA and CUR-PLGA blends significantly increases from 0.00099 at 25 °C to 0.610 at 85 °C and from 0.00019 at 25 °C to 0.566 at 85 °C, respectively, so the use of elevated temperature is recommended for such systems (solubility is increased by 4 orders of magnitude); see Figure S1 in Supporting Information. Moreover, if PLLA/PLGA blend is used as the host material for CUR, it is recommended to introduce as high as possible amount of PLLA because solubility of curcumin in PLLA is greater than in PLGA for all temperature ranges. For RES, such effects (i.e., increase of the maximal molar ratio for RES in the polymer matrix) are also observable, although they are less spectacular (0.003 at 25 °C and 0.028 at 85 °C in the case of both polymers). In contrast to CUR and RES, MMC comprises numerous labile chemical motifs; therefore, the use of elevated temperature is pointless—MMC is very prone to degradation under such conditions, which is well attested in the literature.

In summary, the use of elevated temperatures is highly recommended for curcumin since the solubility of CUR in the polyester matrix is ca. 4 orders of magnitude greater at 85 °C than in 25 °C. In order to conduct the process successfully, the nanocarriers’ preparation at elevated temperature should be followed by rapid cooling to room temperature in order to “freeze” the polymer blend with CUR and avoid unwanted crystallization of the polymer or the payload. On the other hand, this effect is significantly smaller (ca. 1 order of magnitude between 25 and 85 °C) for RES; therefore, encapsulation at higher temperatures may be beneficial, although it is not necessary. Conversely, a MMC colloidal dispersion should be prepared at room temperature to avoid drug degradation.

Our investigations enabled us to find out the optimal conditions for samples’ preparations. For all samples, the dosing rate of the organic phase was equal to 0.139 mL/min (0.114 g/min for CUR-loaded system and 0.109 g/min for RES- and MMC-loaded systems)—see systems CUR2, RES2, and MMC2 in Table 2. CUR-loaded systems were obtained utilizing an elevated (85 °C) process temperature, followed by rapid cooling, while RES- and MMC-loaded systems were prepared at room (25 °C) temperature.

#### 3.1.4 FT-IR and pH-Metric Control of the Batch Process

One step of any drug delivery system preparation method comprising spontaneous emulsification of one phase into another is the removal of the organic solvent. Typically, for volatile organic solvents, e.g., acetone, dichloromethane, chloroform, tetrahydrofuran, or ethanol, this step is performed by evaporation (at room temperature, under reduced pressure, at elevated temperature, by stripping with inert gas, etc.; sometimes two or more of the aforementioned conditions are combined). Conversely, organic solvents with high boiling points, such as dimethyl sulfoxide or *N*,*N*-dimethylformamide, are most often removed by dialysis. The process of solvent removal is rarely controlled; typically, procedures involve “stirring at room temperature for 24 h”, “stirring under reduced pressure for at least a few hours”, etc.^10,31−36^ Sometimes the amount of residual organic solvent in the final colloidal system is determined analytically to determine whether the solvent can be administered safely. On the other hand, to optimize technological processes, it is necessary to know exactly when the organic solvent concentration is below appropriate levels.

Our studies comprise a technologically useful method of organic solvent removal: stripping with inert gas (N_2_). Every 10 min after the completion of the organic phase, a sample was taken and analyzed via FT-IR. A carbonyl stretch (1716 cm^–1^) was used as an indicator of the presence of organic solvent in the colloidal dispersion—see Figures S2–S4 in Supporting Information. Not surprisingly, organic solvents were removed most rapidly from systems loaded with CUR (after 30 min there were no detectable signals attributed to acetone) due to the elevated processing temperature (85 °C). For the core–shell nanoparticles loaded with RES or MMC, 80 or 90 min at room temperature, respectively, was needed to completely remove acetone. These results clearly show that the production costs of drug delivery systems may be significantly reduced by simple stripping with nitrogen with mixing—there is no need for extended evaporation of the organic solvent.

To achieve an appropriate pH for the process mixture in the vessel, it was possible to pump 0.1 M aqueous NaOH while maintaining a constant pH. The pH was set to 7.0 ± 0.5, taking into account the possible acidification of the mixture by gradual hydrolysis of ester moieties in “building blocks” (both HF-PEs and polyesters are prone to hydrolysis). In general, the pH of the mixture was stable—the highest amount of sodium hydroxide was introduced into the mixture of core–shell nanoparticles loaded with RES—at approximately 5 mL. Most likely, this effect is connected to the acidic character of the carboxylic acid end groups in PES oligomers. This phenomenon—the formation of additional amphipathic compounds in our system—may involve additional stabilizing factors responsible for electrostatic repulsion. For samples loaded with CUR and MMC, the amounts of introduced sodium hydroxide were significantly lower (approximately 1–2 mL), corresponding to a low amount of ionizable end groups in both PLLA and PLGA.

### 3.2 Payload Location in the Core–Shell Compartments by XPS and AFM

In general, any microscopic technique can provide appropriate information about the nanocarrier shapes and dimensions. On the other hand, it is difficult to directly analyze the chemical composition of core–shell nanoparticles or gain proof of the location of guest molecules within polymeric or inorganic matrices.^37−42^ Moreover, it should be taken into consideration, that core–shell entrapment may utilize physical interaction, a chemical reaction (especially polymerization or grafting), or their combination so the structure of final nanocarriers is determined by the applied preparation techniques.^43−46^ Another complication is that typically, the preparation of samples prior to electron microscopy (either scanning or transmission), especially for soft matter samples, requires treatment such as sputtering with gold or the adsorption of heavy-metal-containing contrast agents, which completely changes the chemical structure. Therefore, AFM and XPS were both used to enable full analysis of the external and internal structures of nanoparticles, including the payload locus and sample stability upon dilution. Moreover, it should be noted that typical and previously described techniques,^4,9,10,20^ used for core–shell nanocarriers analysis, cannot provide valuable information concerning the actual structure and surface composition. Such emergently missing information, needed for optimal process design, are gained in our studies by XPS with Ar^+^ sputtering (so-called “depth profiling”), followed by appropriate signal processing and analysis as well as supported by AFM for samples’ morphologies.^47,48^ It should be emphasized that AFM and XPS are completely compatible since, in contrast to SEM or TEM, they can be used without direct interaction with the sample. Therefore, we can study both the morphology and chemical composition of the sample directly after drying. The depth profiling by Ar^+^ sputtering, although destructive, may be utilized with combination of the known sample in order to find the evidence of core–shell structure on the basis of the differences of heavy atoms’ (i.e., atoms heavier than hydrogen) abundance in internal and external layers of the sample.^47,48^

First of all, AFM images revealed that the nanoparticles were round in shape and not prone to aggregate. In general, both the diluted and concentrated samples exhibited no tendency to aggregate, most likely due to strong electrostatic repulsion. In general, all of the images showed rough spherical objects of ca. 100–300 nm in length, corresponding to the data from DLS (with hydrodynamic diameters of ca. 120–200 nm and low or moderate PdIs).

**Figure 2:**
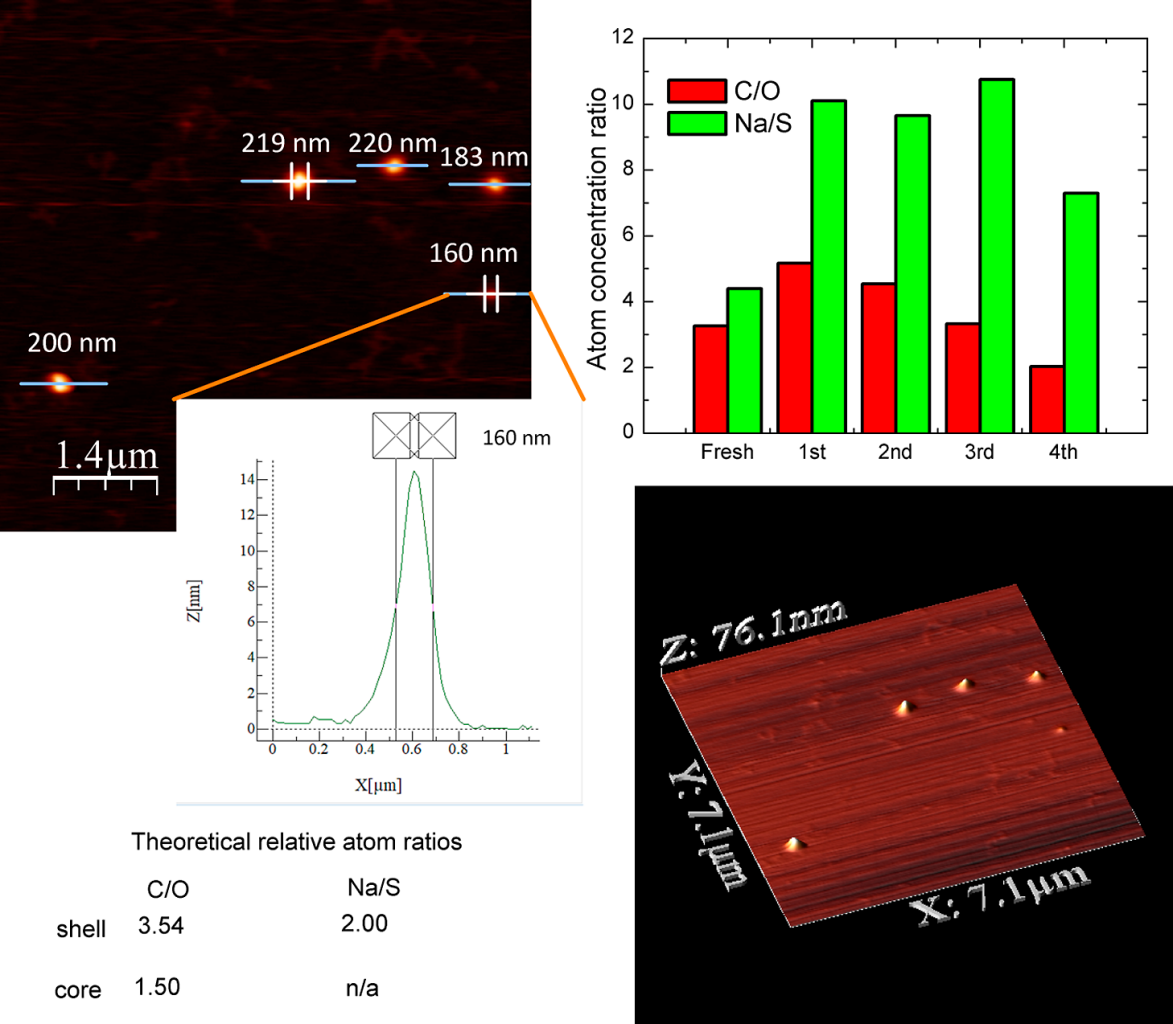
General overview of the CUR-loaded system (CUR2 in Table 2): AFM image of the sample on mica plate (left up) with a profiled single nanoparticle (center), 3D view (left down), theoretical relative atom ratios for core and shell structures (left down), and results of XPS analysis with ion sputtering (right up).

**Figure 3:**
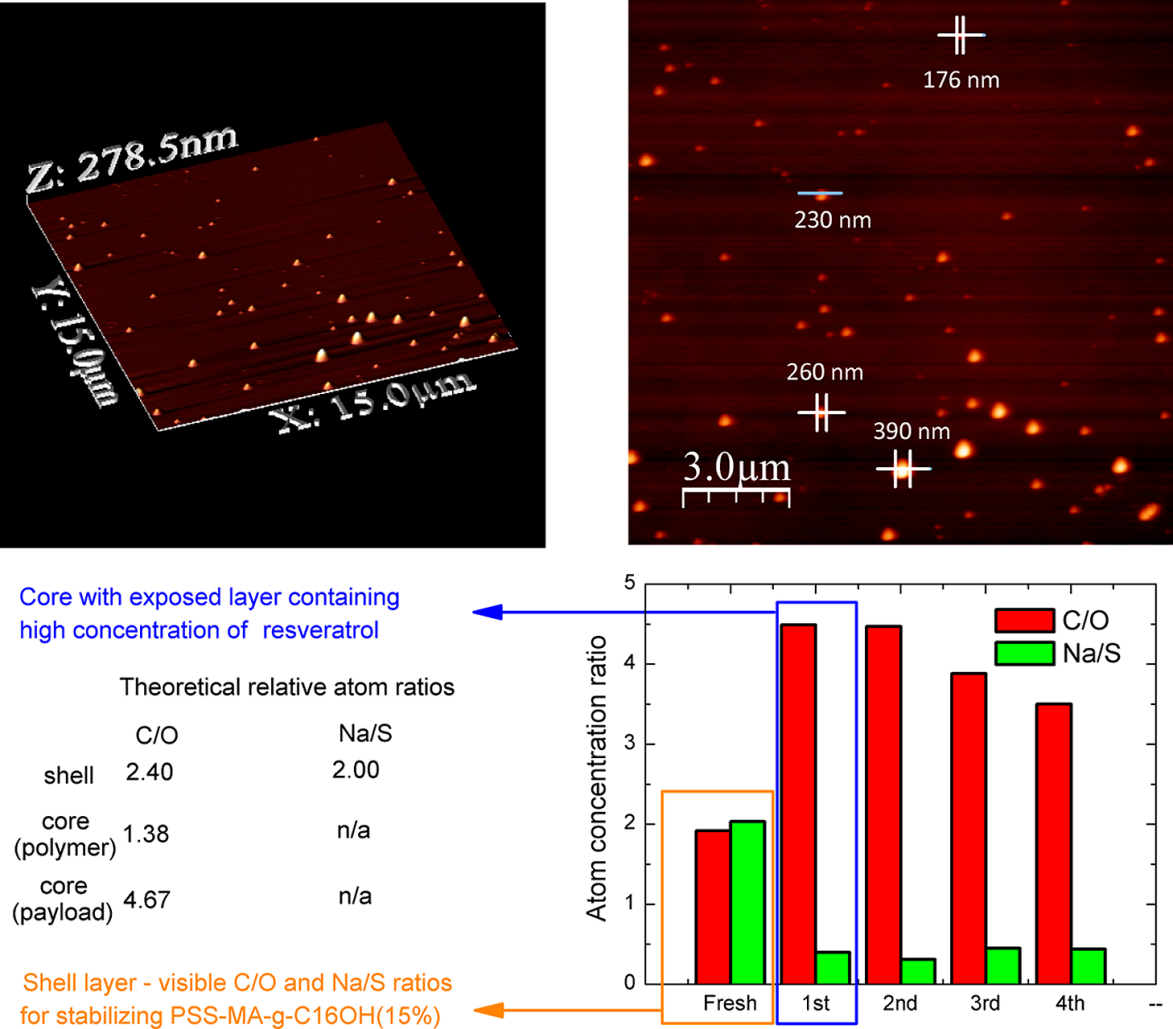
General overview of the RES-loaded system (RES2 in Table 2): 3D AFM image of the sample on mica plate (left up), AFM image with measured core–shell nanoparticles (right up), theoretical relative atom ratios for core and shell structures (left down), and results of XPS analysis with ion sputtering (right down).

**Figure 4:**
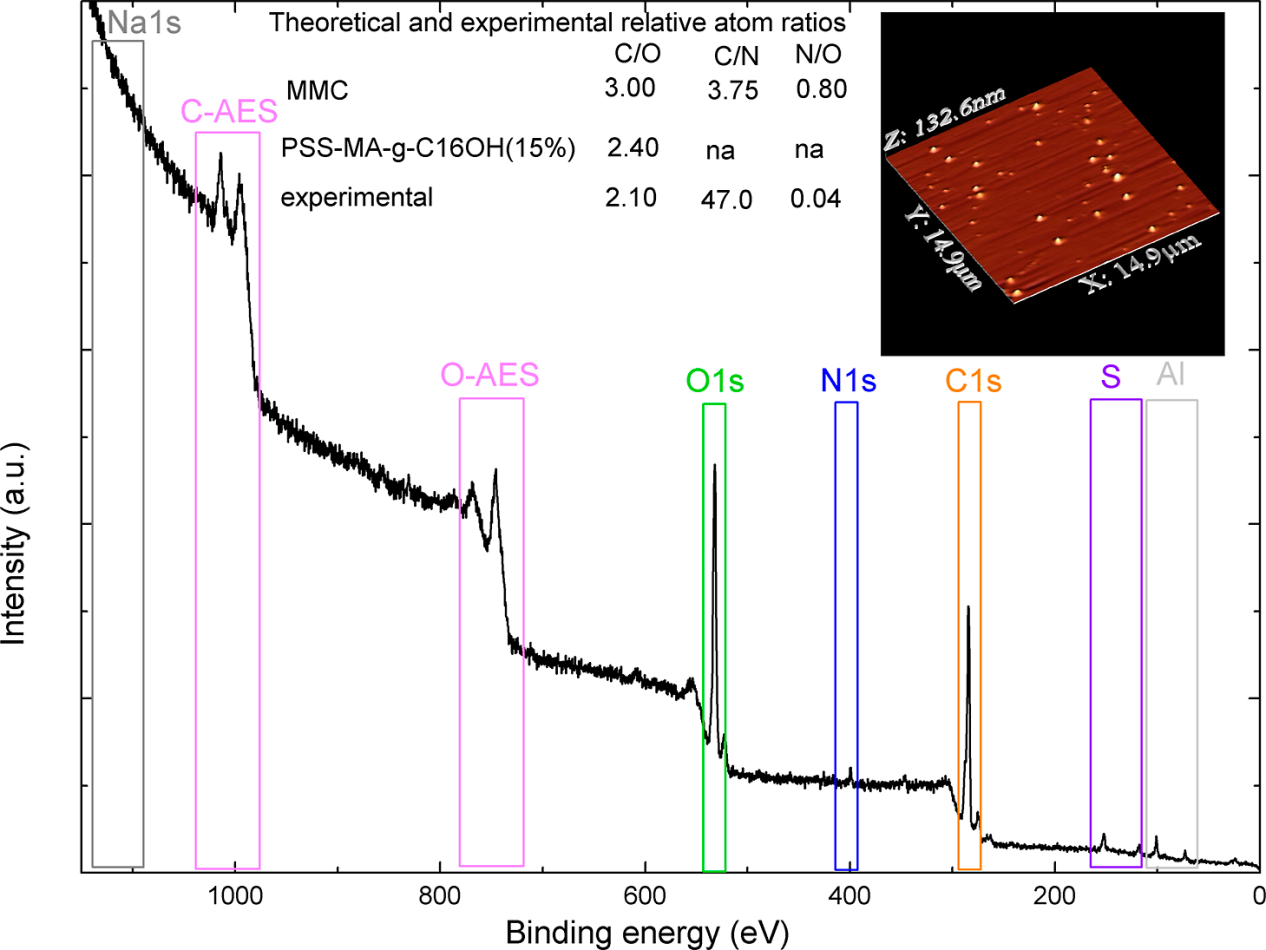
General overview of the MMC-loaded system (MMC2 in Table 2): XPS survey scan showing abundance of carbon, oxygen, nitrogen, sulfur, sodium, and aluminum atoms in the shell layer as well as C and O Auger signals, theoretical and experimental atom ratios, and 3D AFM image of the sample (inset).

In order to study the chemical structure of core–shell nanoparticles, we used a nondestructive XPS method together with profiling as well as Ar^+^ sputtering. In general, analysis of shell layer for all systems (see CUR2, RES2, and MMC2) was performed utilizing nondestructive techniques. It should be emphasized, that, typically, XPS gathers signals from the surface up to a depth of 10 nm although around 90% of the data come from the most external layer of 1 nm.^41^ For all samples, signals, attributed to the presence of carbon, oxygen, sulfur, and sodium, were detected prior to execution of any ion sputtering experiments. This finding indicates the presence of HF-PEs in the shell layer of nanoparticles. Moreover, careful studies of their atomic surface abundance enabled to calculate relative ratios of particular elements (see Figure 2 for the CUR-loaded system, Figure 3 for the RES-loaded system, and Figure 4 for the MMC-loaded system). The calculated values of C/O and Na/S for CUR- and RES-loaded nanoparticles were in very good agreement with the theoretical atomic concentrations, derived from the known chemical structures, and enabled to distinguish the difference between PSS-MA-*g*-C_16_OH(40%) – stabilizing agent for CUR-loaded nanoparticles and PSS-MA-*g*-C_16_OH(15%), used in fabrication of RES-loaded nanocarriers. For MMC-loaded systems, signals, attributed to carbon (ca. 280–292 eV), nitrogen (ca. 398–406 eV), and oxygen (ca. 528–538 eV) atoms, were detected, indicating that MMC is present not only within the core but also adsorbed at the surface. Taking into account the relative abundances: C/O, C/N, and N/O (see Figure 4) we could estimate that the surface concentration of MMC is around 5% so is equal to theoretical value for whole dry mass of nanoparticles (see Table 1). Therefore, we can conclude that MMC is, most possibly, equally present in nanoparticles’ cores and shells.

The chemical composition determined by XPS can provide data about not only the elements and their composition but also their form, i.e., chemical bonds. Therefore, we can estimate the ratios of particular elements as well as carbonyl, sp^3^, sp^2^, or sp^1^ carbon. To analyze the internal structure, ion sputtering can be used to expose the cores of the nanoparticles, although such a destructive method may provide significant changes in the chemical structure. Typically, signals attributed to carbon or oxygen atoms comprise a few peaks with integrated intensities dependent on the abundance of particular type of chemical bonds. The most common ones, like C=O (carbonyl), C–O, or C=C, are characterized by known value of binding energy, accurately determined and tabulated,^41,47,48^ for both ranges of C (around 280–292 eV) and O (around 528–538 eV) atoms. Therefore, fitting (deconvolution) of the experimental peaks with multiple Gauss curves of the fixed values on the X axis (binding energy) may enable us to find the relative abundance of particular motifs. The exemplified spectra with fitting are shown in Figures S5–S9 while calculated values of relative abundances of particular bonds (C=O, C–C, and C=C), both experimental and theoretical, are shown in Tables S4–S6. The relative abundances of particular elements’ species are provided in Table S7. Our studies are in good agreement with the known structures (see Table 1) of shell-forming material, i.e., HF-PEs. For all the analyzed samples, the most indicative was the C=O/C–C ratio, due to abundance of both carbon species in the shell in both PSS-MA-*g*-C_16_OH(40%), theoretical value of 0.12 and PSS-MA-*g*-C_16_OH(15%), theoretical value of 0.24. The experimentally assessed ratios were in good agreement with theoretical ones: 0.14 [sample CUR2—see Table S4, stabilized by PSS-MA-*g*-C_16_OH(40%)] and 0.21 [samples RES2—see Table S5 and MMC2—see Table S6, stabilized by PSS-MA-*g*-C_16_OH(40%)], indicating, that the shell layer comprise practically exclusively HF-PEs. Moreover, for sample RES2, there were detected double carbon–carbon bonds, most possibly connected with presence of resveratrol molecules adsorbed at the interface between the core and the shell; note that XPS collects a signal up to depth of ca. 10 nm. Such an effect, increased the concentration of resveratrol at the interface, is most probably connected with the worst compatibility (when compared with CUR and MMC; see subchapter 3.1.1) of RES with a polyester matrix, and XPS proved the chemical stability of such a payload form. Similarly, as in the case of atom abundances ratios, Ar^+^ sputtering drastically destroyed the structure and sequential procedures led to stabilization of particular bond ratios. Therefore, such a procedure, ion sputtering, cannot provide reliable data for the exposed core layers, especially for fragile bonds like double carbon–carbon or carbonyl motifs.

XPS and ion sputtering, combined with compatible AFM, can provide unique data, confirming the core–shell structure of the studied carriers systems. We can not only prove that HF-PE is present at the shell layer but also prove stability of the payload, when possible.

## 4 Conclusions

The design of core–shell nanocarriers for the amphiphilic drugs CUR, RES, and MMC, characterized by a high increase in dispersion and hydrogen bonding, involved careful studies of both the process (dosing rates, temperature, and pH) and solubility (calculated utilizing HSPiP software) parameters as well as PAT approaches (online FT-IR measurements, AFM and XPS with Ar^+^ sputtering investigation of the final colloids to assess the payload locus) in batch-type vessels. First, the processes have been studied in terms of solubility parameters and qualitative descriptors [components of dispersion (δ_d_), polar interactions (δ_p_), and hydrogen bond interactions (δ_h_); maximal molar ratios, when possible] to determine the optimal host material for CUR-PLLA plasticized by PLGA, RES-PLGA plasticized by PES and MMC-pure PLGA. All the systems were stabilized utilizing a newly designed class of compounds—HF-PEs—with very high potential to adsorb at interfaces. Considering the solubility parameter, CUR-loaded core–shell nanoparticles were prepared at a higher temperature (85 °C) than those of RES and MMC (room temperature). To prevent hydrolysis of the payload and host material, the pH was maintained at 7 ± 0.5 by the addition of controlled amounts of 0.1 M NaOH. Only in the case of RES-loaded particles was a significant amount of aqueous sodium hydroxide introduced (ca. 5 mL), most likely to neutralize terminal acidic groups of oligomeric plasticizers. Timely sampling and FT-IR analysis enabled us to determine how long it was necessary to perform stripping of N_2_ to completely remove the organic solvent.

The obtained colloidal core–shell nanoparticles were characterized by mean diameters of approximately 120–200 nm and low polydispersity. Moreover, the stability of the core–shell nanoparticles was very high due to electrostatic repulsion, as the zeta potential ranged from −65 to −85 mV. The optimal, for all of the studied systems, organic phase dosing rate was approximately 0.139 mL/min; introducing the organic phase too fast or too slow resulted in the formation of larger and more polydisperse samples. It should be noted that the dimensions obtained via DLS were confirmed by AFM; additionally, it was proven that sample dilution did not influence the diameter of the particles. XPS with Ar^+^ sputtering confirmed the core–shell structure of the nanoparticles, and sulfur and sodium, connected with the responsible for sample stabilization HF-PEs, were located in the external layer of the sample. Signals attributed to sp^2^ carbon (double bonds) were found for samples loaded with RES before ion sputtering and are, most probably, connected with the presence of payload molecules in a layer just below the shell. These findings confirmed the location of the payload in the core of the nanoparticles as well as indicated, that the shell layer is very thick, although can sufficiently stabilize nanocarriers and protect the payload.

Our studies revealed the parameters needed to optimize the core–shell entrapment of bioactive compounds, namely, the dosing rate, temperature, time needed to completely remove organic solvents by inert gas stripping, pH control, and composition. Careful analysis by XPS with Ar^+^ sputtering, especially of the payload locus within polymeric compartments, enabled us to confirm our predictions by theoretical and experimental methods and to validate the optimized parameters.

## Supporting Information Available

Studies of solubility parameter difference between the studied polymers/oligomers and the payloads; dependence of CUR and RES solubility in the polymer matrix on temperature; organic solvent removal studied by FT-IR; XPS scans with fitting for ion sputtering; and element species concentration determination (PDF)
