# Supplementary material for: Entrapment of Amphipathic Drugs in Core–Shell Polymeric Nanoparticles under Batch Conditions—The Role of Control and Solubility Parameters
Source: Langmuir. 2024 Sep 24;40(40):21186–98. doi: 10.1021/acs.langmuir.4c02721 (PMC11465662; doi:10.1021/acs.langmuir.4c02721)
Supplement: Supplementary file 1 — la4c02721_si_001.pdf [file la4c02721_si_001.pdf]

ELECTRONIC SUPPLEMENTARY MATERIAL

Entrapment of amphipathic drugs in core–shell polymeric nanoparticles under batch conditions: – the role of control and solubility parameters.

Łukasz Lamch [1,*], Rafał Szukiewicz [2]

[1.] Department of Engineering and Technology of Chemical Processes, Faculty of Chemistry, Wrocław University of Science and Technology, Wybrzeże Wyspiańskiego 27, 50-370 Wrocław, Poland

[2.] Faculty of Physics, University of Wrocław, M. Borna 2, 50-383 Wrocław, Poland

* Corresponding author.

Email addresses:

lukasz.lamch@pwr.edu.pl

1. *Studies of solubility parameter difference between the studied polymers / oligomers and the payloads.*

Table S1. Solubility parameter difference (Δδ, see equation 2 in main body) calculated for δ_d, δ_p and δ_h values obtained by Y-MB method.

				CUR	RES	MMC	PLLA	PES	PLGA
			δ_h	*20.1*	*20.9*	*19.7*	*16.1*	*18.0*	*15.9*
			δ_p	*8.1*	*6.7*	*11.6*	*13.3*	*13.4*	*14.8*
	δ_d	δ_p	δ_h	*11.3*	*13.1*	*10.8*	*4.9*	*5.2*	*5.5*
CUR	*20.1*	*8.1*	*11.3*	0.00	2.42	3.56	**9.17**	**8.35**	**9.81**
RES	*20.9*	*6.7*	*13.1*	2.42	0.00	5.54	**11.57**	**10.76**	**12.18**
MMC	*19.7*	*11.6*	*10.8*	3.56	5.54	0.00	**7.12**	**6.12**	**7.26**
PLLA	*16.1*	*13.3*	*4.9*	9.17	11.57	7.12	**0.00**	**1.93**	**1.63**
PES	*18.0*	*13.4*	*5.2*	8.35	10.76	6.12	**1.93**	**0.00**	**2.54**
PLGA	*15.9*	*14.8*	*5.5*	9.81	12.18	7.26	**1.63**	**2.54**	**0.00**

Table S2. Solubility parameter difference (Δδ, see equation 2 in main body) calculated for δ_d, δ_p and δ_h values obtained by Hoftyzer-van Krevelen's method.

				CUR	RES	MMC	PLLA	PES	PLGA
			δ_d	18.9	19.3	18.6	15.6	17.7	16.0
			δ_p	4.4	4.8	7.9	8.6	6.6	7.2
	δ_d	δ_p	δ_h	15.2	18.0	13.8	11.1	11.5	12.0
CUR	*18.9*	*4.4*	*15.2*	0.00	2.94	3.82	**8.83**	**4.93**	**7.19**
RES	*19.3*	*4.8*	*18.0*	2.94	0.00	5.40	**10.81**	**7.47**	**9.24**
MMC	*18.6*	*7.9*	*13.8*	3.82	5.40	0.00	**6.62**	**3.20**	**5.55**
PLLA	*15.6*	*8.6*	*11.1*	8.83	10.81	6.62	**0.00**	**4.67**	**1.85**
PES	*17.7*	*6.6*	*11.5*	4.93	7.47	3.20	**4.67**	**0.00**	**3.49**
PLGA	*16.0*	*7.2*	*12.0*	7.19	9.24	5.55	**1.85**	**3.49**	**0.00**

Table S3. Solubility parameter difference (Δδ, see equation 2 in main body) calculated for δ_d, δ_p and δ_h values obtained by Stefanis-Panayiotou's method.

				CUR	RES	MMC	PLLA	PES	PLGA
			δ_d	*17.3*	*17.9*	*18.7*	*16.9*	*17.7*	*17.2*
			δ_p	*13.1*	*10.3*	*15.0*	*9.9*	*10.8*	*12.9*
	δ_d	δ_p	δ_h	*23.2*	*28.9*	*10.4*	*7.9*	*8.7*	*8.8*
CUR	*17.3*	*13.1*	*23.2*	0.00	6.38	13.02	**15.64**	**14.69**	**14.40**
RES	*17.9*	*10.3*	*28.9*	6.38	0.00	19.10	**21.03**	**20.21**	**20.28**
MMC	*18.7*	*15.0*	*10.4*	13.02	19.10	0.00	**5.96**	**4.64**	**3.04**
PLLA	*16.9*	*9.9*	*7.9*	15.64	21.03	5.96	**0.00**	**1.45**	**3.15**
PES	*17.7*	*10.8*	*8.7*	14.69	20.21	4.64	**1.45**	**0.00**	**2.16**
PLGA	*17.2*	*12.9*	*8.8*	14.40	20.28	3.04	**3.15**	**2.16**	**0.00**

2.*Dependence of CUR and RES solubility in polymer matrix on temperature.*

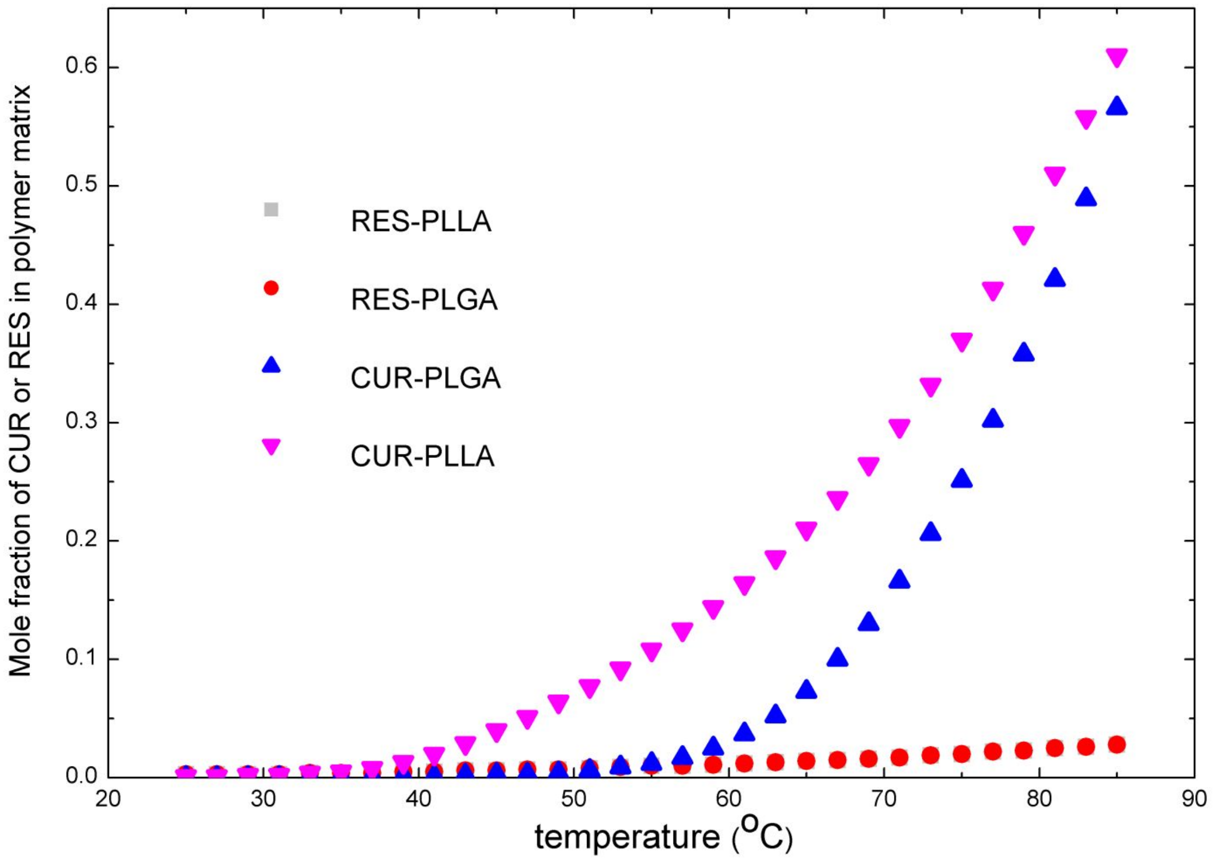

Fig. S1. Maximal mole fraction of CUR and RES in polymer matrix (PLLA, PLGA) in dependence of temperature.

3.*Organic solvent removal studied by FT-IR*

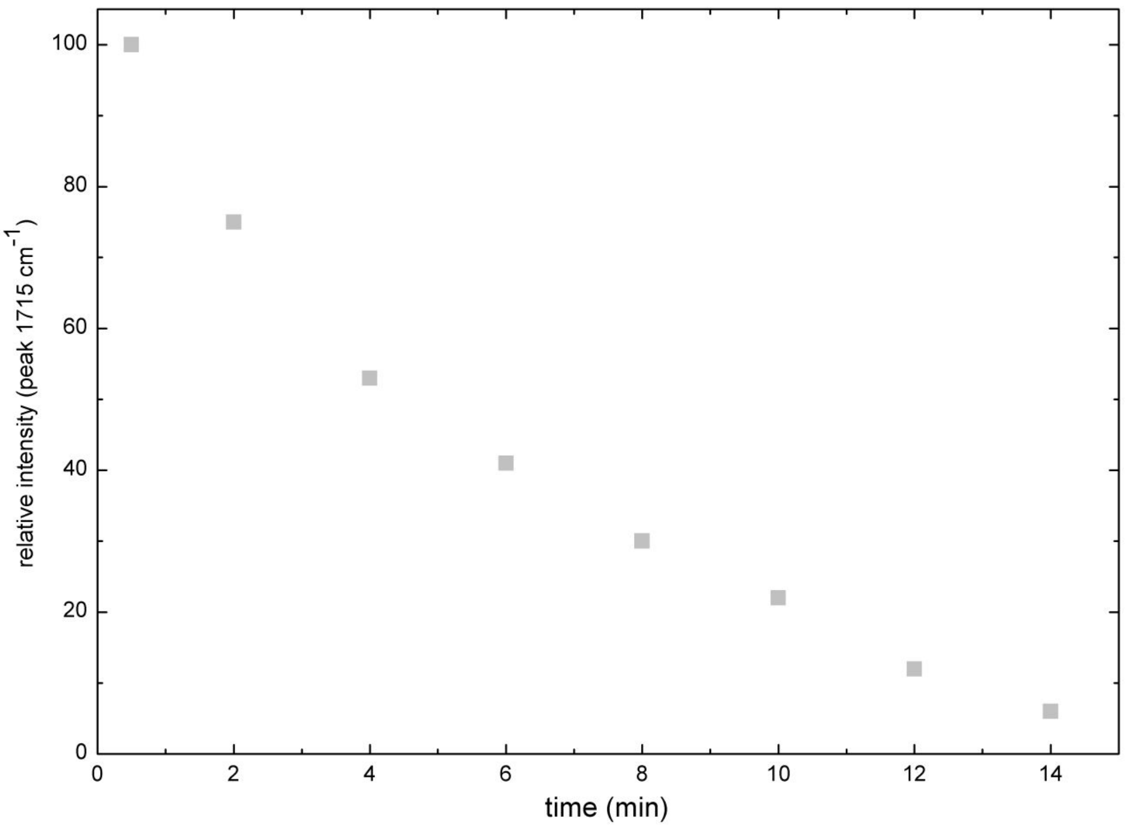

Fig. S2. Time-dependant FT-IR spectra of CUR-loaded systems.

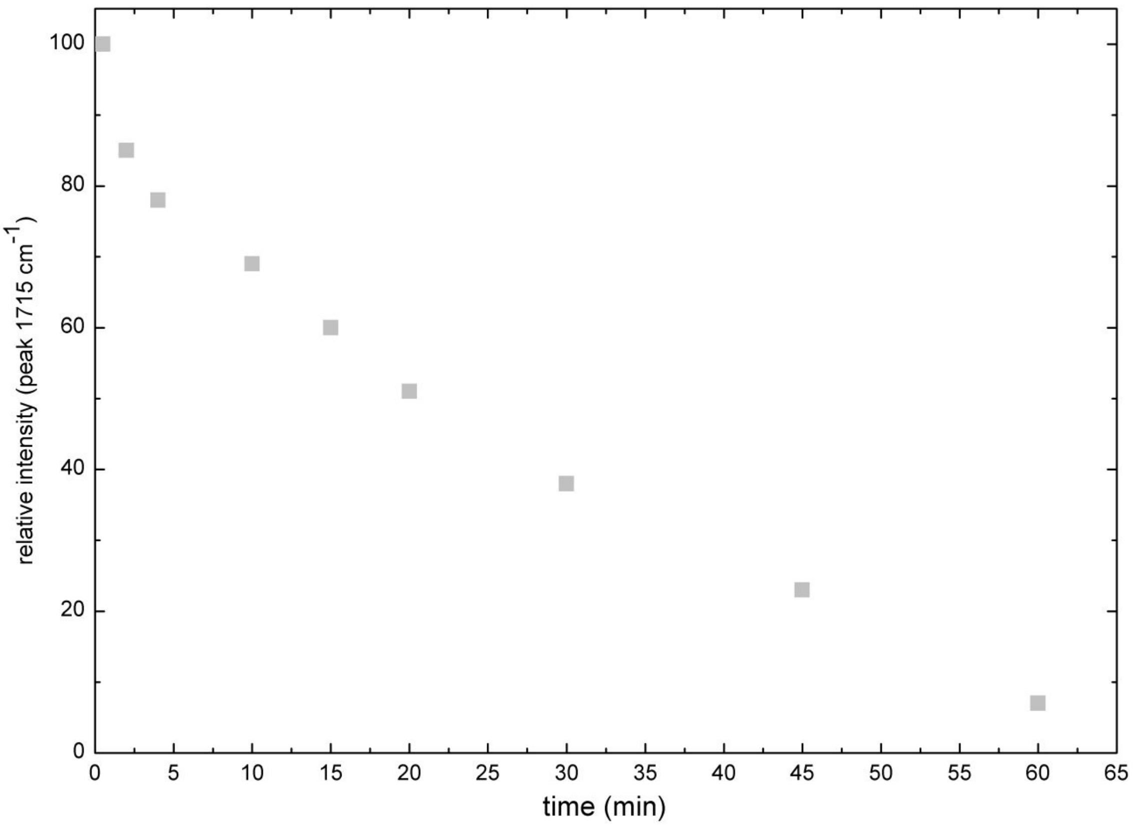

Fig. S3. Time-dependant FT-IR spectra of RES-loaded systems.

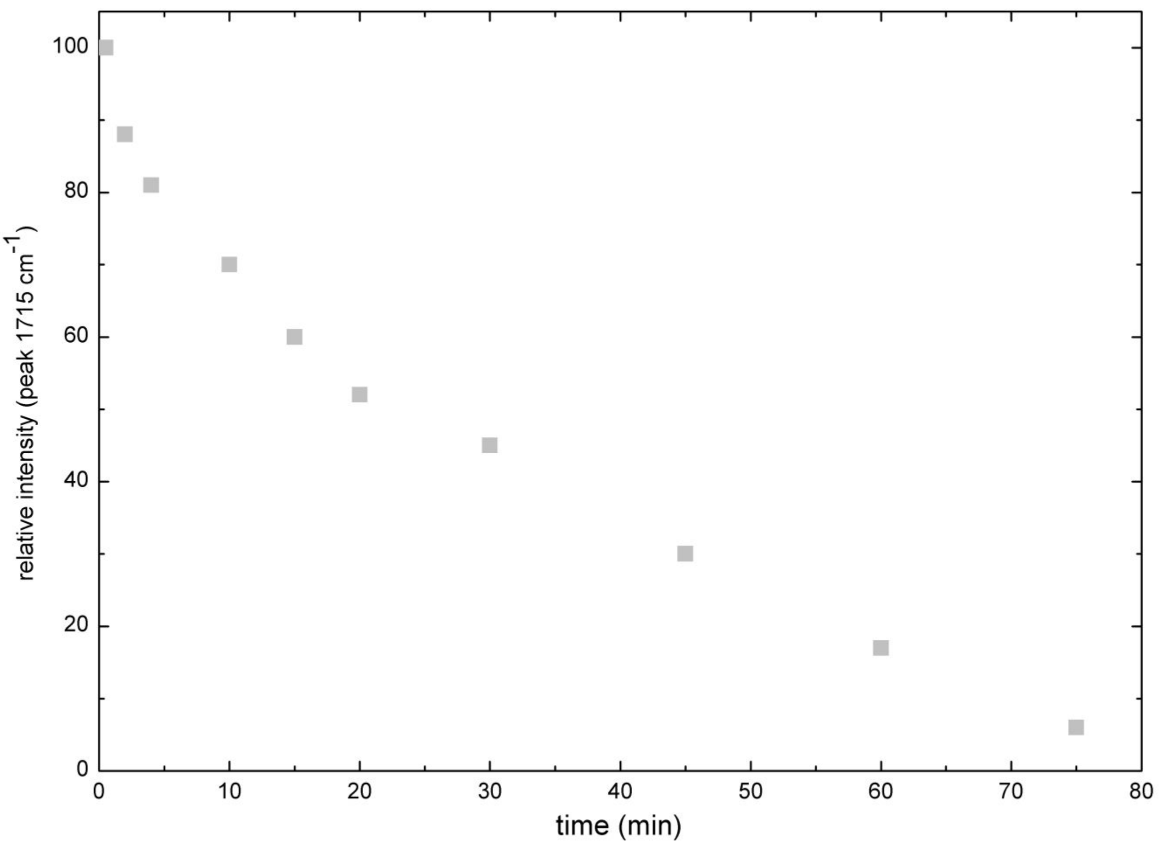

Fig. S4. Time-dependant FT-IR spectra of MMC-loaded systems.

4.*XPS scans with fitting for ion sputtering and elements species concentrations determination*

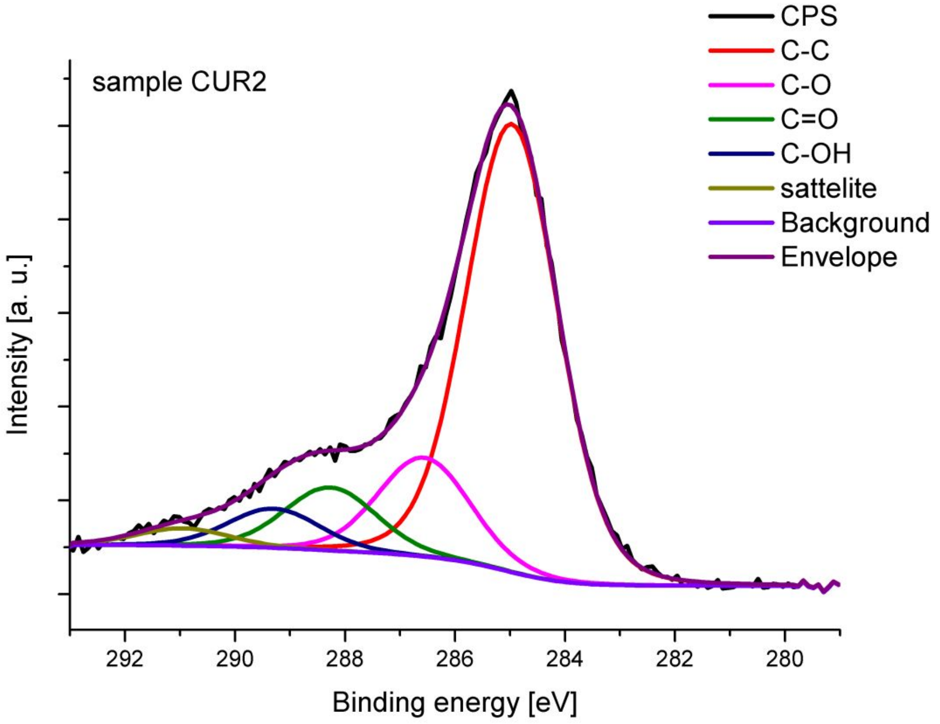

Fig. S5. XPS spectra for sample CUR2 (see Table 2) with fitting for different carbon atom species before ion sputtering.

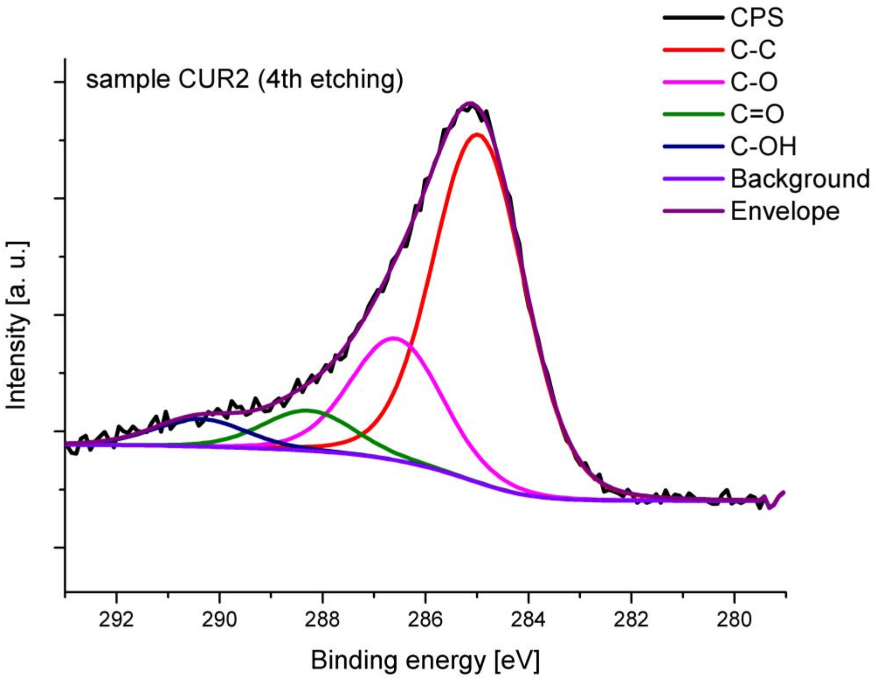

Fig. S6. XPS spectra for sample CUR2 (see Table 2) with fitting for different carbon atom species after 4th session of ion sputtering.

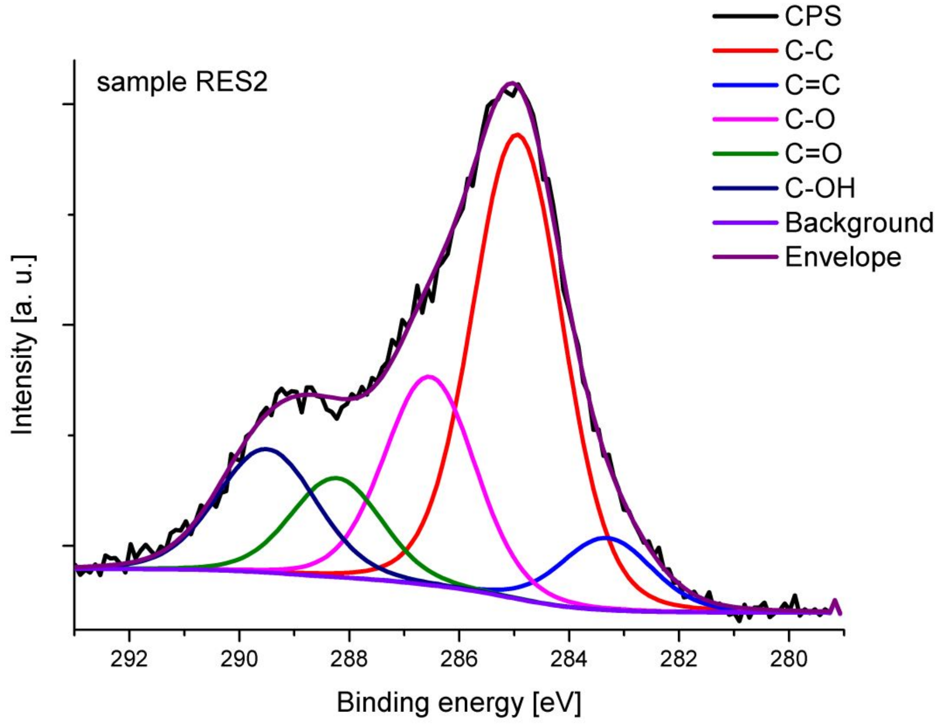

Fig. S7. XPS spectra for sample RES2 (see Table 2) with fitting for different carbon atom species before ion sputtering.

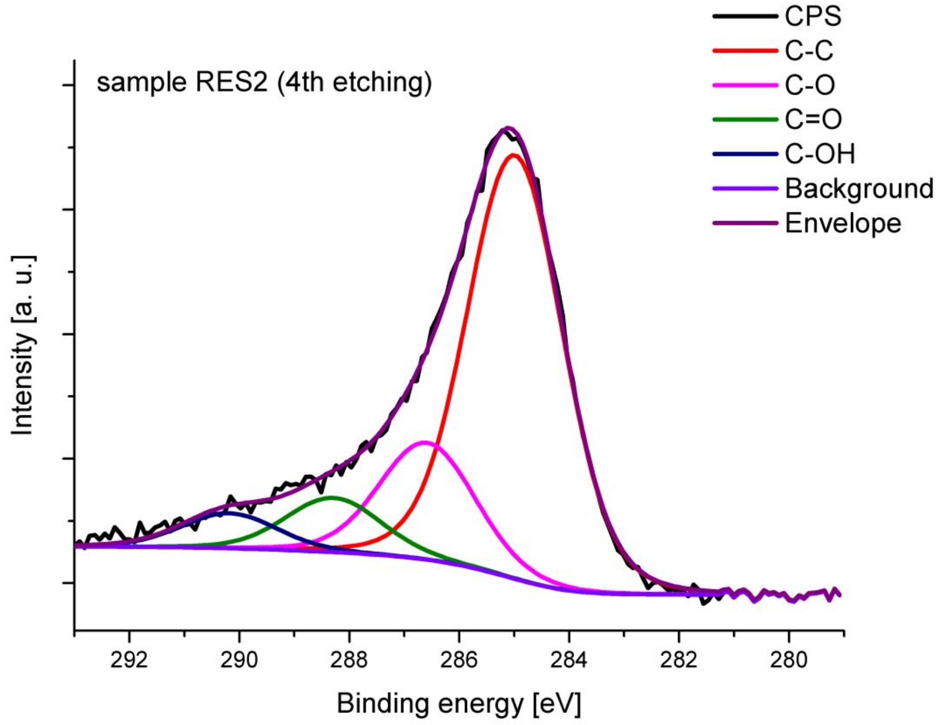

Fig. S8. XPS spectra for sample RES2 (see Table 2) with fitting for different carbon atom species after 4th session of ion sputtering.

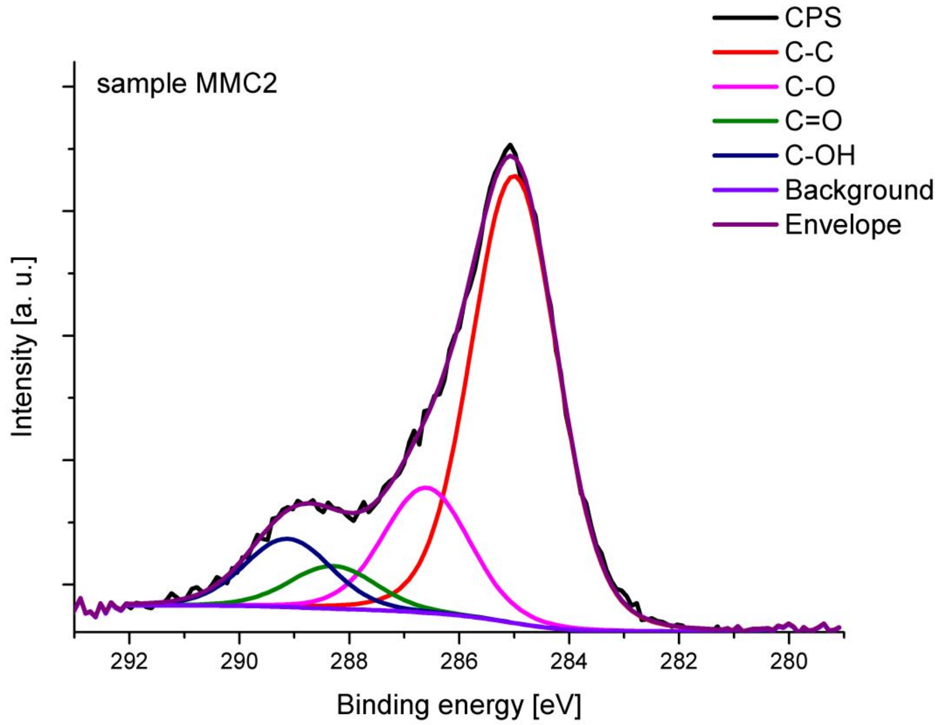

Fig. S9. XPS spectra for sample MMC2 (see Table 2) with fitting for different carbon atom species.

Table S4. The relative ratio of carbonyl (C=O) to aliphatic (C-C) bonds in CUR2 sample:

Fresh	1st	2nd	3rd	4th	core (theoretical)	shell (theoretical)
0.14	0.12	0.12	0.11	0.12	1.34	0.12

Table S5. The relative ratio of carbonyl (C=O) and double carbon-carbon (C=C) to aliphatic (C-C) bonds in RES2 sample:

	Fresh	1st	2nd	3rd	4th	core (theoretical)	shell (theoretical)
C=O/C-C	0.21	0.13	0.12	0.13	0.13	1.00	0.24
C=C/C-C	0.16	0.00	0.00	0.00	0.00	0.39	0.00

Table S6. The relative ratio of carbonyl (C=O) and double carbon-carbon (C=C) to aliphatic (C-C) bonds in MMC2 sample:

	Fresh (experimental)	MMC (theoretical)	PSS-MA-g-C_{16}OH(15%) (theoretical)
C=O/C-C	0.21	0.33	0.24
C=C/C-C	0.00	0.00	0.00

Table S7. The relative abundance of the selected elements' species at the samples' surfaces prior any ion sputtering sequences.

atom specimen	CUR2 sample (atom%)	RES2 sample (atom%)	MMC2 sample (atom%)
C	68.98	62.21	62.05
O	21.18	32.46	29.61
S	1.34	0.61	1.22
Al	0.00	2.73	5.50
N	2.63	0.75	1.32
Na	5.87	1.24	0.30
